# Distribution of Periodontal Pockets Among Smokers and Nonsmokers in Patients with Chronic Periodontitis: A Cross-sectional Study

**DOI:** 10.7759/cureus.5586

**Published:** 2019-09-06

**Authors:** Surekha Velidandla, Ruparani Bodduru, Vinod Birra, Yash Jain, Rathna Valluri, Kranti Kiran Reddy Ealla

**Affiliations:** 1 Oral and Maxillofacial Pathology, MNR Dental College and Hospital, Hyderabad, IND; 2 Periodontology, MNR Dental College and Hospital, Hyderabad, IND; 3 Dentistry, Government Medical College, Srikakulam, IND; 4 General Dentistry, Malla Reddy Institute of Dental Sciences, Hyderabad, IND

**Keywords:** bleeding on probing, periodontal pocket depth, periodontitis, smoking

## Abstract

Context

Cigarette smoking is a well-established risk factor for periodontitis and carries an increased risk for loss of periodontal attachment as well as bone loss.

Aims

The purpose of the present study was to investigate whether disease severity differs between smokers and nonsmokers in a group of chronic periodontitis patients by assessing the periodontal probing depth (PPD) and bleeding on probing (BOP).

Materials and methods

The study included 150 individuals, 75 smokers and 75 nonsmokers, in the age group of 35-60 years. Subjects with chronic periodontitis were selected and included in the study. Periodontal evaluation, including periodontal probing pocket depths and bleeding on probing, was performed on all four quadrants and at six sites per tooth using the Williams periodontal probe. The data were pooled from the anterior sextant and the posterior sextant as well as from the facial and lingual surfaces.

Statistical analysis

Comparisons were made between smokers and nonsmokers using the z-test (two-tailed test). Probing pocket depth categories 0-3 mm, 4-5 mm, 6-7 mm, and ≥8 mm and the proportion of sites having a pocket depth of ≥5 mm were used in the analysis.

Results

The mean percentage of sites that bleed upon probing was higher for nonsmokers as compared with smokers. Smokers had less shallow pockets (0-3 mm) than nonsmokers and more pockets of 4-7 mm (categories 4-5 mm, 6-7 mm). No significant differences were detected in the prevalence of pockets ≥8 mm. In the anterior, premolar, and molar regions, pockets of 6-7 mm were significantly more prevalent in smokers. The buccal and lingual sides also showed that smokers had more sites with deep probing depths ≥5 mm than nonsmokers. The data also showed that in the upper jaw, in the anterior and premolar teeth, the largest differences were found between smokers and nonsmokers.

Conclusions

From the results, it can be concluded that cigarette smoking results in periodontal tissue destruction in the different areas of the oral cavity, with the maximum periodontal destruction in the maxillary anterior and premolar region.

## Introduction

Periodontitis is the result of complex interrelationships between infectious agents and host factors. Environmental, acquired, and genetic risk factors modify the expression of disease and may, therefore, affect the onset or progression of periodontitis [1]. Among the environmental risk factors, tobacco smoking has been found to be associated with an increased prevalence and severity of periodontal disease [2]. It is also apparent that a disproportionately high number of people with severe periodontal disease are smokers [3] and that a strong association exists between smoking and an unusual form of periodontitis that is resistant to treatment.

In a study evaluating the effect of nonsurgical treatment in smokers and nonsmokers, the degree of pocket reduction was significantly lower in smokers. The strongest difference was observed for pockets of the maxillary anterior region [4], a finding also suggestive of local effects. Furthermore, this local effect is also substantiated by the observation that smokers, in general, have proportionately more periodontal pocketing in the anterior segments than those who have never smoked [2,5]. The purpose of the present study was to investigate whether the disease severity differs between smokers and nonsmokers in a group of chronic periodontitis patients.

## Materials and methods

The present study was conducted in the department of periodontology, MNR Dental College and Hospital, Sangareddy, Hyderabad, India. The sample size of the study was 150 individuals, with 75 smokers, and 75 nonsmokers in the age group of 35-60 years. Subjects with chronic periodontitis were selected and included in the study. Periodontal evaluation, including periodontal probing pocket depths and bleeding on probing, was performed on all four quadrants and at six sites per tooth using the Williams periodontal probe. Patients who were systemically healthy with chronic periodontitis and smokers who smoke ≥10 cigarettes daily for ≥10 years were included in the study. Patients who underwent periodontal therapy and who were on antibiotic therapy were excluded.

For both parameters (probing pocket depth, bleeding on probing), full mouth mean scores were calculated, as well as scores considering the upper jaw, lower jaw, buccal, lingual, anteriors, premolars, and molars. Probing pocket depth categories 0-3 mm, 4-5 mm, 6-7 mm, and ≥8 mm and the proportion of sites having a pocket depth of ≥5 mm were used in the analysis. Comparisons were made between smokers and nonsmokers using the z-test (two-tailed test).

## Results

Mean age, mean number of teeth, and mean percent of sites did not differ between smokers and nonsmokers (Table 1). The mean percent of sites that bleed upon probing was higher for nonsmokers as compared to smokers. Smokers had less shallow pockets (0-3 mm) than nonsmokers and more pockets (4-7 mm; categories 4-5 mm, 6-7 mm). No significant differences were detected in the prevalence of pockets ≥8 mm. In the anterior, premolar, and molar regions, pockets of 6-7 mm were significantly more prevalent in smokers (Table 2). The overall differences in the prevalence of probing depths ≥5 mm between smokers and nonsmokers were 48% and 37%, respectively (Table 3). In the upper jaw, 48% of the sites in smokers were ≥5 mm while 37% of the sites in non-smokers showed probing depths ≥5 mm. The buccal and lingual sides also showed that smokers had more sites with deep probing depths ≥5 mm than nonsmokers. The data also showed that in the upper jaw, in the anterior and premolar teeth, the largest differences were found between smokers and nonsmokers. Smokers had proportionally more pockets ≥ 5 mm, especially on the palatal and lingual surfaces (Table 4).

**Table 1:** Number of teeth, mean % of sites showing bleeding upon probing by location, and % oral distribution of probing pocket depth (PPD) categories, presented by smoking status; standard deviation in parentheses. z-test between nonsmokers and smokers; ns represents there is no significant difference between the two variables; ** represents a significant difference between two variables at a 5% level of significance

	Nonsmokers	Smokers	z-test
n	75	75	
Age (years)	47 (8)	46 (6)	ns
Mean # of teeth	25 (3.6)	25.7 (3.8)	ns
Anterior	11.1 (1.5)	11.1 (1.2)	ns
Premolars	7.7 (1.6)	7.9 (1.7)	ns
Molars	7.5 (2.8)	7.2 (2.8)	ns
% distribution of probing pocket depth (PPD) categories			
%PPD 0-3mm	48.6 (19.5)	36.8 (5.9)	**
%PPD 4-5mm	37.7 (12.9)	39.3 (12.3)	ns
%PPD 6-7mm	15.2 (8.2)	22.2 (10.2)	**
%PPD ≥8 mm	9.2 (6.5)	10.2 (5.6)	ns

**Table 2:** Percent oral distribution of probing pocket depth categories presented by smoking status (nonsmokers, n=75/smokers, n=75); standard deviation in parentheses z-test between non smokers and smokers; ns represents there is no significant difference between two variables; ** represents a significant difference between two variables at a 5% level of significance

	Nonsmokers	Smokers	z-value
Anterior			
%PPD 0-3mm	56 (22)	41 (19)	**
%PPD 4-5mm	31 (15)	38(16)	**
%PPD 6-7mm	10 (12)	16 (15)	**
%PPD ≥8 mm	5 (7)	6(9)	ns
premolars			
%PPD 0-3mm	44(18)	34(15)	**
%PPD 4-5mm	38(14)	40(15)	ns
%PPD 6-7mm	14(8)	20(13)	**
%PPD ≥8 mm	6(8.5)	7(9)	ns
Molars			
%PPD 0-3mm	22(14.5)	15(9.5)	**
%PPD 4-5mm	42(17)	43(16)	ns
%PPD 6-7mm	20(15)	35(14)	**
%PPD ≥8 mm	18(14.5)	14(12)	ns

**Table 3:** Percent of sites with a probing pocket depth ≥5 mm presented by location and by smoking status (nonsmokers, n=75/smokers, n=75); standard deviation in parentheses z-test between nonsmokers and smokers; ns represents there is no significant difference between the two variables; ** represents there is a significant difference between the two variables at the 5% level of significance

	Nonsmokers	Smokers	z-value
All sites	37 (19)	48 (17)	**
Anterior	25 (20)	34(22)	**
Premolars	35(23)	45(21)	**
Molars	54(21)	63(16)	**

**Table 4:** Percent of sites with a probing pocket depth ≥5 mm presented by location and by smoking status standard deviation in parentheses z-test between nonsmokers and smokers; ns represents there is no significant difference between the two variables; ** represents there is a significant difference between the two variables at the 5% level of significance

	Nonsmokers		Smokers	z-value
Upper jaw				
All sites	35(20)		46(18)	**
Anterior	26(24)		39(24)	**
Premolars	38(27)		51(21)	**
Molars	56(22)		65(18)	**
Lower jaw				
All sites	30(20)		40(19)	**
Anterior	23(22)		34(21)	**
Premolars	29(24)		37(22)	**
Molars	54(23)		65(19)	**
Buccal				
All sites	30(19)		41(18)	**
Anterior	24(22)		35(22)	**
Premolars	29(22)		36(21)	**
Molars	54(25)		60(19)	**
Lingual				
All sites	39(22)		49(20)	**
Anterior	25(24)		35(24)	**
Premolars	38(25)		55(23)	**
Molars	58(23)		67(19)	**
Upper jaw buccal				
All sites	40(24)		55(22)	**
Anterior	30(30)		45(30)	**
Premolars	43(28)		57(24)	**
Molars	59(26)		66(22)	**
Lower jaw buccal				
All sites	30(22)		39(28)	**
Anterior	22(24)		33(25)	**
Premolars	24(26)		29(22)	**
Molars	49(27)		60(23)	**
Lower jaw lingual				
All sites	35(22)		44(21)	**
Anterior	18(27)		26(23)	**
Premolars	35(27)		50(31)	**
Molars	61(28)		67(26)	**
Upper jaw palatal				
All sites	42(26)	56(24)		**
Anterior	31(30)	44(28)		**
Premolars	44(31)	57(24)		**
Molars	57(27)	68(24)		**

Data were further analyzed by dividing the smokers into two subgroups based on cigarette consumption. The mean proportion of pockets ≥5mm was higher for those who smoked ≥ 20 cigarettes per day than those who smoked 11-20 cigarettes.

## Discussion

The present study investigated whether cigarette smoking affects the extent of pocketing and bleeding on probing in chronic periodontitis patients. Bleeding upon probing (BOP) provides a quantitative indication of gingival/periodontal inflammation. BOP acts as diagnostic predictability, as bleeding on probing and loss of attachment are interrelated [6]. Smokers presented with reduced signs of inflammation as compared to nonsmokers, which is expressed on bleeding on probing [7-8]. The decreased bleeding among smokers can be attributed to the temporary gingival vasoconstriction induced by nicotine. It is also suggested that swollen gingiva in smokers represent decreased vascularity density and angiogenesis as compared to nonsmokers, which is due to the suppressed inflammatory response [9]. Cigarette smoke comprises many components that can alter the function of immune cells [10-11].

Smokers had fewer sites with bleeding on probing as compared with nonsmokers. Most investigators had found that smokers had less bleeding on provocation than nonsmokers, although one study reported smokers had more bleeding [2,12]. Another study reported no differences were found between smokers and nonsmokers [13]. Decreased gingival bleeding in smokers had been explained as being due to nicotine, which causes vasoconstriction of peripheral blood vessels such as in the forearm, skin, and hands [14].

Nicotine can cause a dose-dependent inhibition on type 1 collagen production and fibronectin production. There is an increase in the collagenase activity, which equals to an approximately 700% and 400% increase per 0.075% and 0.05% of nicotine concentration [15].

Several studies had shown a relationship between the number of cigarettes smoked and the prevalence and severity of periodontitis [16-20]. Patients smoking more than 10 cigarettes per day or smoking more than 10 years were relatively more frequent in the moderate to advanced periodontitis group, whereas those smoking less or for a shorter duration were not [5]. Smoking impairs chemotaxis, decreases phagocytosis by neutrophils, and reduces antibody production, resulting in detrimental effects on the periodontium [21-22]. The effect of vasoconstriction and decreased oxygen tension create a compatible subgingival environment for the colonization of anaerobic bacteria [23]. Many studies have proved the pattern of periodontal destruction among smokers, considering the local effect of smoking on the palatal surfaces or on the maxillary anterior region [24-26]. The difference in patterns of pocketing in smokers and nonsmokers was suggestive of a local effect. The present study showed that the proportion of pockets ≥5 mm did differ in almost all instances. Data were separated by upper and lower jaw, buccal and lingual surfaces, and tooth type. In the upper jaw, in the anterior and premolar teeth, the largest differences were found between smokers and nonsmokers. Smokers have more sites with pocket depth ≥5 mm, especially on the lingual surface of these teeth. The probing depth categories showed that smokers had primarily less shallow pockets and more pockets between 4 mm and 7 mm. The difference between smokers and nonsmokers was most pronounced in the anterior region. These observations correlate with earlier findings [5,13] and were suggestive of a local effect.

## Conclusions

In conclusion, the present study indicates that cigarette smoke has many components associated with deeper periodontal pockets in association with impaired immunological response. The pocket severity also follows an intraoral distribution pattern, which is related to the local effect of smoke, altering the local temperature and favoring plaque formation. The implications of this are that efforts at smoking cessation should be considered in the treatment of periodontitis. Furthermore, prevention and counseling should be part of community education for the purpose of preventing periodontal diseases. By acquiring tobacco intervention skills, dentists, hygienists, and assistants can take a leading role among health professionals in having a significant impact on the negative health effects associated with the use of tobacco products.

## References

[1] Page RC, Offenbacher S, Schroeder HE, Seymour GJ, Kornman KS (2007). Advances in the pathogenesis of periodontitis: summary of developments, clinical implications and future directions. Periodontol 2000.

[2] Haber J, Wattles J, Crowley M, Mandell R, Joshipura K, Kent RL (1993). Evidence for cigarette smoking as a major risk factor for periodontitis. J Periodontol.

[3] Bergstrom J (1989). Cigarette smoking as risk factor in chronic periodontal disease. Community Dent Oral Epidemiol.

[4] Preber H, Bergstrom J (1986). The effect of non-surgical treatment on periodontal pockets in smokers and non-smokers. J Clin Periodontol.

[5] Haber J, Kent RL (1992). Cigarette smoking in a periodontal practice. J Periodontol.

[6] Al-Bayaty FH, Baharuddin NA, Abdulla MA, Mohd Ali H, Arkilla MB, ALBayaty MF (2013). The influence of cigarette smoking on gingival bleeding and serum concentrations of haptoglobin and alpha 1-antitrypsin. Biomed Res Int.

[7] Haffajee AD, Socransky SS (2001). Relationship of cigarette smoking to attachment level profiles [Article in German]. J Clin Periodontol.

[8] Müller HP, Heinecke A, Eger T (2000). Site-specific association between supragingival plaque and bleeding upon probing in young adults. Clin Oral Investig.

[9] Bergström J, Persson L, Preber H (1988). Influence of cigarette smoking on vascular reaction during experimental gingivitis. Scand J Dent Res.

[10] Sopori M (2002). Effects of cigarette smoke on the immune system. Nat Rev Immunol.

[11] Adamson J, Thorne D, McAughey J, Dillon D, Meredith C (2013). Quantification of cigarette smoke particle deposition in vitro using a triplicate quartz crystal microbalance exposure chamber. Biomed Res Int.

[12] Newbrun E (1996). Indices to measure gingival bleeding. J Periodontol.

[13] van der Weijden GA, de Slegte C, Timmerman MF, van der Velden U (2001). Periodontitis in smokers and non-smokers: intra-oral distribution of pockets [Article in German]. J Clin Periodontol.

[14] Larson PS, Silvette H (1961). Tobacco. Experimental and Clinical Studies (Supplement III).

[15] Chahal GS, Chhina K, Chhabra V, Chahal A (2017). Smoking and its effect on periodontium - revisited. Indian J Dent Sci.

[16] Grossi SG, Genco RJ, Machtei EE (1995). Assessment of risk for periodontal disease. II. Risk indicators for alveolar bone loss. J Periodontol.

[17] Martinez-Canut P, Lorca A, Magán R (1995). Smoking and periodontal disease severity. J Clin Periodontol.

[18] González YM, De Nardin A, Grossi SG, Machtei EE, Genco RJ, De Nardin E (1996). Serum cotinine levels, smoking, and periodontal attachment loss. J Dent Res.

[19] Krall EA, Dawson-Hughes B, Garvey AJ, Garcia RI (1997). Smoking, smoking cessation, and tooth loss. J Dent Res.

[20] Tomar SL, Asma S (2000). Smoking-attributable periodontitis in the United States: findings from NHANES III. J Periodontol.

[21] Persson L, Bergstrom J, Ito H, Gustafsson A (2001). Tobacco smoking and neutrophil activity in patients with periodontal disease. J Periodontol.

[22] Palmer RM, Wilson RF, Hasan AS, Scott DA (2005). Mechanisms of action of environmental factors - tobacco smoking. J Clin Periodontol.

[23] Salvi GE, Lawrence HP, Offenbacher S, Beck JD (1997). Influence of risk factors on the pathogenesis of periodontitis. Periodontol 2000.

[24] Haber J (1994). Smoking is a major risk factor for periodontitis. Curr Opin Periodontol.

[25] Axelsson P, Paulander J, Lindhe J (1998). Relationship between smoking and dental status in 35-, 50-, 65- and 75-year-old individuals. J Clin Periodontol.

[26] van der Weijden GA, de Slegte C, Timmerman MF, van der Velden U (2001). Periodontitis in smokers and nonsmokers: intra-oral distribution of pockets [Article in German]. J Clin Periodontol.

